# Editorial

**Published:** 1864-10

**Authors:** 


### Editorial.

CHICAGO MEDICAL SOCIETY.—This Society continues to prosper in our city. We are glad to see that most of the well-educated young men who locate in the city, promptly seek for membership, and are cordially received. With the commencement of October, the weekly meetings of the Society were resumed, and have been well attended. Though the meetings as heretofore conducted are interesting and profitable, yet we think there is room for improvement, especially in the manner of discussing important questions in pathology and practice. Instead of restricting each member to *ten minutes* in the discussion, for the purpose of allowing all the members opportunity to speak during the same evening, it would be more interesting and profitable to have those who speak on any subject occupy time enough to develop all the facts concerning it, and if necessary let the same topic be continued for two or three evenings in succession. The ten minutes rule leads to a disultory conversation, without the thorough discussion of anything.

CHICAGO MEDICAL COLLEGE.—The fifth annual course of instruction, in this institution, commenced on Monday evening, October 10. The general introductory lecture was delivered by Dr. M. O. Heydock, Professor of Medical Jurisprudence and Lecturer on Materia Medica.

The lecture room of the college was well filled, chiefly with medical students and practitioners. The evident object of the lecturer, was to strongly impress upon the minds of the class the necessity and advantages, of patient application and untiring industry in the prosecution of their studies. The lecture was well written, and listened to by the audience with pleasure and profit. The number of students in attendance is one-third larger than during any previous year. The hospital is also well filled with patients, laboring under acute diseases and idiopathic fevers, affording ample facilities for true clinical instruction. We take pleasure in thus chronicling the steady progress of both the college and the hospital, for they are fully entitled to the confidence and support of the profession.

A New Ambulance.—Assistant-Sugeon Howard, of the U. S. Army, has prepared a model for an improved Ambulance, for use in the army.

Promotion.—Surgeon Richard S. Satterlee, of U. S. Army, has been promoted to the rank of Brigadier-General.

Surgeon-General of U.S.A.—Dr. Joseph K. Barnes has recently been appointed Surgeon-General of United States Army, in the place of Dr. Wm. A. Hammond, removed.

Explanation.—We shall hereafter continue to issue the Examiner without trimming. Almost all our subscribers wish to have it bound in a volume at the end of each year; and experience has fully shown that the trimming of each number, as it is issued, so frequently cuts the pages irregularly that it injures the volume, for binding, when completed.

Symptoms of Poisoning Following a Dose of Disulpate of Quinine.—Sir, I beg to forward the accompanying case, as I think it may prove interesting to many members of the profession:—

I was called in great haste one evening to a case of supposed poisoning. On arriving at the house, I found the man much better, but thought it safer to administer an emetic. On making enquiries, I found that he had prescribed for himself a mixture of sarsaparilla and quinine, which he had purchased from a druggist. The symptoms of poisoning came on after the first dose of this mixture: within a few minutes there was severe pain and burning in the stomach; the face swelled; the mouth felt drawn forwards; then the legs and body swelled, and became very red, with intolerable itching, followed by a rash of urticaria. I thought it possible that some poison had become accidentally mixed with his mixture, and so the case rested for a time. However, shortly afterwards the man had an attack of pneumonia, and during his convalescence quinine was prescribed. Upon taking the first dose (two-thirds of a grain, Howard's), all the symptoms above described came on, clearly proving that the quinine was the original cause of the mischief.

I think this case interesting from the singularity and violence of the symptoms produced by so small a dose, and also as showing how a druggist may be unjustly blamed from an idiosyncrasy of a patient. I am, sir, your obedient servant,

*Bradford, Feb.* 1864.—*London Lancet.* E. H. Roe.

---

Uterine Action During Sleep.—Sir, I have read with great interest Dr. Palfrey's case in reference to the above subject, and, in confirmation of his opinion, beg, to forward you the following statement.

I was called on the morning of the 20th of October last, to see a lady in her second confinement. Her residence being within a few doors of my own, no time was lost in visiting her. I found the child's head resting upon the perineum, and in a few minutes she was safely delivered of a fine healthy son. On going to bed the previous night she felt quite well, and had no intimation of the event about to take place, except a slight discharge, of which she took no great notice.

In this case, there is no doubt that the whole stage of dilatation, and partly that of expulsion, had taken place during sleep, as from her awaking until the birth of the child no longer period than half an hour could have elapsed. Her first confinement, some twelve months previously, had been very severe, occupying some forty-eight hours, with delivery by forceps. In this instance there were only three or four labor pains, and no after-pains whatever. I am, Sir, your obedient servant,

John Harvey, M.D.

*Cheltenham*, 1864.—*London Lancet.*

---

In the Vomiting of Pregnancy.—Dr. Kroyher, of Presburg, considers the tincture of nux vomica a specific. He directs a few drops to be taken in a little aromatic or cherry-laurel water, increasing it to ten, twelve, or eighteen drops, if necessary, every morning early, and in the evening. *Br.* 3–156.

A Forearm Wrenched off during Efforts at Reduction for Dislocation.—M. Adolphe Guerin related to the Paris Surgical Society, of which he is a member, the following extraordinary case. A woman aged sixty-three was admitted into the St. Louis Hospital for a luxation of the shoulder-joint of three months' standing. On examination, it was found that the humeral head was lying under the coracoid process; and, notwithstanding the long time the dislocation had existed, M. Guerin immediately gave directions for an attempt at reduction. Not having pulleys at hand, mere traction was tried first. Extension was made by a lac, first fixed above the elbow, and afterwards at the wrist. Counter-extension was employed as usual, and four very intelligent and steady pupils were desired to pull gently, steadily, and without jerking. Whilst traction was thus gradually being accomplished, a snapping noise was heard, and the forearm fell to the ground, the operators, rather terrified, being besprinkled with blood from a spouting artery. M. Guerin controlled the vessel immediately, applied a ligature, pared the wound, sawed off the protruding portion of the humerus, covered the wound with a lateral flap farmed by the tearing process, and obtained a stump of the usual kind. By examining the forearm lying on the ground, it was found that the severance had taken place at the elbow, and the forearm seemed to have broken off like a dead bough breaks off from a tree. The bones and surrounding parts were soft and friable, and the muscles could easily be unravelled with the finger, like a clot of blood. All the textures, in short—vessels, nerves, muscles, and bone, were discovered to be unsound, and the radius and ulna were snapped across by the moderate traction which the students had employed. Microscopic examination confirmed these views. M. Guerin considered that the alterations above alluded to were the result of the compression of the brachial plexus during the luxated state of the head of the humerus; and he thought that the practical lesson to be derived from this accident was, that surgeons having to treat luxations of three months' standing will do well to order tractions of a very moderate kind, so as to avoid so unpleasant an issue as the one just described. It seems to us that the *age* of the woman had a good deal to do with the result; nor can it be overlooked that the pulling of four persons at an aged patient's arm was in itself a prodedure of some peril.—*London Lancet.*

---

Paraplegia.—Dr. Brown Séquard says that nux vomica should be avoided as a most dangerous poison, in all cases of paraplegia in which there are signs of congestion or inflammation of the spinal cord or its meninges, for in these it but increases the cause of the paralysis, and produces an aggravation of the symptoms. He says there are two distinct groups of cases of paraplegia, one distinguished by symptoms of irritation, the other characterized by the absence of them. The symptoms of irritation observed in the former class are convulsions, cramps, twitchings, erection of the penis, formication, and itching; diminution of temperature, wasting of the muscles, œdema, bed sores, and alkaline urine. In the second class all these symptoms are wanting, and the paraplegia is caused by the white or non-inflammatory softening, or is of the reflex kind; for this class nux vomica is particularly applicable, from the power it possesses of augmenting the amount of blood sent to the spinal cord and membranes, and, from the extra nutrition thereby derived, of increasing the vital properties of this nervous centre. —*Braithwaite* 43–26.

